# Individual Tree Crown Segmentation and Crown Width Extraction From a Heightmap Derived From Aerial Laser Scanning Data Using a Deep Learning Framework

**DOI:** 10.3389/fpls.2022.914974

**Published:** 2022-06-14

**Authors:** Chenxin Sun, Chengwei Huang, Huaiqing Zhang, Bangqian Chen, Feng An, Liwen Wang, Ting Yun

**Affiliations:** ^1^School of Information Science and Technology, Nanjing Forestry University, Nanjing, China; ^2^Research Institute of Forest Resource Information Techniques, Chinese Academy of Forestry, Beijing, China; ^3^Danzhou Investigation and Experiment Station of Tropical Crops, Ministry of Agriculture, Rubber Research Institute, Chinese Academy of Tropical Agricultural Sciences, Danzhou, China; ^4^College of Forestry, Nanjing Forestry University, Nanjing, China

**Keywords:** airborne LiDAR, deep learning, heightmap, individual tree crown segmentation, forest parameter retrieval

## Abstract

Deriving individual tree crown (ITC) information from light detection and ranging (LiDAR) data is of great significance to forest resource assessment and smart management. After proof-of-concept studies, advanced deep learning methods have been shown to have high efficiency and accuracy in remote sensing data analysis and geoscience problem solving. This study proposes a novel concept for synergetic use of the YOLO-v4 deep learning network based on heightmaps directly generated from airborne LiDAR data for ITC segmentation and a computer graphics algorithm for refinement of the segmentation results involving overlapping tree crowns. This concept overcomes the limitations experienced by existing ITC segmentation methods that use aerial photographs to obtain texture and crown appearance information and commonly encounter interference due to heterogeneous solar illumination intensities or interlacing branches and leaves. Three generative adversarial networks (WGAN, CycleGAN, and SinGAN) were employed to generate synthetic images. These images were coupled with manually labeled training samples to train the network. Three forest plots, namely, a tree nursery, forest landscape and mixed tree plantation, were used to verify the effectiveness of our approach. The results showed that the overall recall of our method for detecting ITCs in the three forest plot types reached 83.6%, with an overall precision of 81.4%. Compared with reference field measurement data, the coefficient of determination *R*^2^ was ≥ 79.93% for tree crown width estimation, and the accuracy of our deep learning method was not influenced by the values of key parameters, yielding 3.9% greater accuracy than the traditional watershed method. The results demonstrate an enhancement of tree crown segmentation in the form of a heightmap for different forest plot types using the concept of deep learning, and our method bypasses the visual complications arising from aerial images featuring diverse textures and unordered scanned points with irregular geometrical properties.

## Introduction

Trees play an important role in the functioning of ecosystems by providing a range of ecological services, such as storing carbon dioxide, preventing flooding and desertification, providing forest habitats, and promoting atmospheric circulation (Liu et al., 2017; Zhang et al., 2019). Acquiring individual tree information is beneficial for forest growth assessment and sustainable forest management (Zhou et al., 2020). Constituting the premise for measuring numerous parameters (e.g., the tree position, height, crown width and distribution density), the effective detection of individual trees using various remote sensing technologies has become one of the primary tasks for precision forestry.

With the rapid growth of remote sensing technology, such as aerial photography, oblique photogrammetry and light detection and ranging (LiDAR), remote sensing has been widely utilized in the acquisition of forest information and land cover data. Moreover, a wide variety of methods have been introduced to process different types of remote sensing data in a range of forest conditions, and they can be divided into two categories. The first category is based on image-processing techniques and computer graphics; these techniques can identify and extract individual tree crowns (ITCs) by directly processing aerial images, heightmaps [i.e., digital surface models (DSMs) or canopy height models] and LiDAR point clouds coupled with image segmentation (Zhou et al., 2020) and point cloud clustering algorithms, to accomplish the recognition or classification of individual trees. Examples of methods in the first category are the marker-controlled watershed method (Hu et al., 2014), graph-cut algorithm (Strîmbu and Strîmbu, 2015), simulation of fishing net dragging (Liu et al., 2015), energy function minimization-based approach (Yun et al., 2021), geometrical feature-driven point cloud merging at the super voxel scale (Ramiya et al., 2019) and trunk location as guidance and point density-based feature employment (Mongus and Žalik, 2015).

The second category for ITC segmentation comprises deep learning-based models for processing unmanned aerial vehicle (UAV) images and forest point clouds. Trees are identified by feeding input UAV images (Lei et al., 2022) and point clouds into multiple conceptual layers using deep learning convolutional neural networks (Zhang et al., 2020a) and tuning the training hyperparameters through a gradient descent strategy, leading to the choices of parameters falling within a reasonable range. These optimization objectives have driven numerous synergetic studies using UAV images and deep learning techniques in forest applications, such as the utilization of U-net (Cao and Zhang, 2020) to map forest types in the Atlantic Forest (Wagner et al., 2019), the employment of DeepLab and an attention domain adaptation network for detecting Amazonian and Southeast Asia palms (Ferreira et al., 2020; Zheng et al., 2020), the adoption of Faster-RCNN for tree seedling mapping (Pearse et al., 2020) and the construction of multitask end-to-end optimized deep neural networks (MEON) for oak and pine detection (Weinstein et al., 2020). Moreover, numerous studies have introduced various deep learning models to process forest point clouds, for example, combining PointNet with point cloud voxelization for ITC segmentation (Chen et al., 2021), proposing a pointwise directional deep embedding network for enhancing the boundaries of instance-level trees (Luo et al., 2021), developing a projection strategy for tree point clouds to generate a set of multiperspective views for various tree species and identify boles using two-dimensional (2D) image processing neural networks (Zou et al., 2017; Hamraz et al., 2019), and using PointNet++ for wood-leaf classification and tree species recognition based on terrestrial laser scanning data (Xi et al., 2020).

Despite the many approaches proposed to segment individual trees from UAV images and LiDAR data, each category has its drawbacks and restrictions. The efficacy of methods based on image processing and computer graphics is usually decreased by the different color or texture appearances of tree crowns constituting the forest plots (Gomes et al., 2018), illumination differences between locally radiant and shaded surfaces causing varying brightness levels within ITCs (Zhou et al., 2020), and overlapping ITCs, which weaken the accuracy of treetop detection and tree crown boundary delineation (Yun et al., 2021). In addition, the efficiency of computer graphics algorithms for ITC extraction is always exacerbated by the geometrical complexity of tree crowns characterized by more apices in the crown periphery and certain conjunctions caused by pendulous and locally protruding branches belonging to the adjacent tree crowns (Hu et al., 2014).

The deep learning-based methods for processing forest UAV images (Xie et al., 2022) and LiDAR data (Hu et al., 2020) also encounter similar sensitivity and susceptibility challenges in tree crown recognition caused by the complexity of forest environments (Qian et al., 2021), image-capture angles (Yin et al., 2021) and interferences stemming from local solar radiation (Kattenborn et al., 2019). Furthermore, the predicted bounding boxes produced by common small-target detection networks, e.g., You Only Look Once (YOLO) and Faster Regional-based Convolutional Neural Network (R-CNN), have regular rectangular shapes, making it difficult to detect the anisotropic shapes of tree crowns. On the other hand, the high dimensional and the unstructured nature of three-dimensional (3D) point clouds mapped the geometrical peculiarity of tree crown periphery, which introduces extreme complications for the segmentation task and makes it difficult to implement deep learning networks with high accuracy (Liu et al., 2020). In addition, many adverse factors, such as mutual occlusions throughout the forest (Zhang et al., 2020b), the need for joint mining of local and global semantic features (Luo et al., 2021) and additional post-treatment for the segmentation results yielded by deep learning networks (Zhang et al., 2020c), need to be considered when using artificial intelligence applications in forestry.

In this work, three novel concepts were proposed to address the above restrictions. First, we transformed aerial laser scanning (ALS) data to heightmaps, which are selected as the data source for the deep learning neural networks. Heightmaps are beneficial for ITC segmentation because these maps avoid the interference encountered in forest aerial images due to variations in solar radiation intensity (Zhang et al., 2022) and texture features induced by different phenological periods of target trees (Zhang and Bai, 2020). In addition, the heightmaps preserve morphological characteristics of the upper tree crowns that reflect a tendency of reduction from treetops to all surrounding areas, and these characteristics are used as salient features to enhance the task of tree crown recognition. Second, to complete data augmentation for obtaining for training samples feed to the deep learning neural networks, three advanced generative adversarial networks (GANs), i.e., the cycle-consistent GAN (CycleGAN), the Wasserstein GAN + gradient penalty (WGAN-GP) and an unconditional GAN trained on a single natural image (SinGAN), were employed to generate synthetic heightmaps of tree crown plot to enhance the recognition capabilities and classification accuracy of the YOLO-v4 deep learning neural network (Bochkovskiy et al., 2020). Third, we adopted a mean shift algorithm instead of a K-means clustering algorithm for adaptive determination of the initial centers of the training sample properties and proposed an elliptic paraboloid fitting method to refine the recognition results of the YOLO-v4 network and determine the point cloud affiliation in the intersecting regions between adjacent bounding boxes with the aim of accurately delineating ITC boundaries with overlapping branches or leaves. Finally, the applicability of the proposed frameworks were verified using various forest plot types, and the calculated ITC width was validated by the values obtained from field measurements.

## Materials and Methods

### Study Site and Data Collection

In this study, three different study sites were investigated, i.e., a tree nursery, forest landscape and mixed forest habitat located at the foot of Nanjing’s Purple Mountain (32.07°N, 118.82°W) and Nanjing Forestry University (32.07°N, 118.78°W), Nanjing, in southeastern China. The city of Nanjing is located south of the Qinling–Huaihe Line, China, and has a subtropical monsoon climate. The annual average temperature is 15.7°C, and the average temperatures in the coldest month (January) and the hottest month (July) are −2.1°C and 28.1°C, respectively. The annual precipitation is 1021.3 mm. The first study site is a tree nursery, where sweet osmanthus (*Osmanthus fragrans* Lour.) and *Acer palmatum* Thunb. have been planted. The trees are arranged in order with a uniform spacing with a relatively small tree crown and lower heights. The second study site is a forest landscape with 3 species of conifers and 23 species of broad-leaved trees, where many dwarf shrubs grow beneath the forest canopy. The third study site is the mixed tree habitat, where 4 species of conifers and approximately 17 species of broad-leaved trees have been planted. The dominant tree species include China fir (*Cunninghamia lanceolata* (Lamb.) Hook.) and *Metasequoia glyptostroboides* Hu & W. C. Cheng.

In October 2019, the Velodyne HDL-32E sensor (Velodyne Lidar, Inc., San Jose, CA, United States) on the DJI FC6310 UAV was used to acquire airborne LiDAR data from the three study sites. The sensor transmits 700,000 laser pulses per second and records the return value of each laser pulse. The horizontal field of view is 360°, and the vertical field of view ranges from +10.67° to –30.67°. The angular resolutions of the sensor are approximately 1.33° (vertical) and 0.16° (horizontal) at 600 revolutions per minute. The beam divergence is approximately 2 mrad with an average footprint diameter of 11 cm. The flight altitude was 60 m with a 15% overlap. In October 2019, aerial photographs of the three study sites were taken using a digital camera mounted on the same UAV flying at a speed of 20 m/s and at an altitude of approximately 100 m.

**FIGURE 1:**
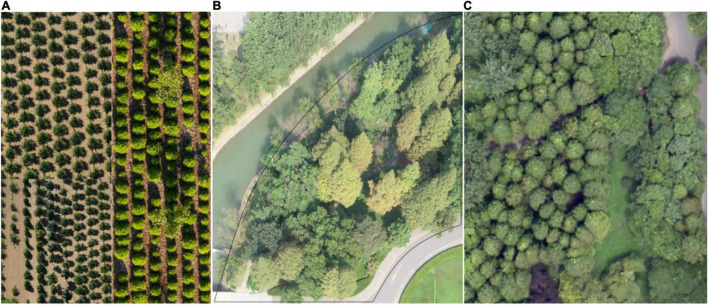
Partial aerial photographs of the studied forest plots. **(A)** The tree nursery at the foot of Nanjing’s Purple Mountain and the **(B)** forest landscape and **(C)** mixed forest habitat on the campus of Nanjing Forestry University.

In October 2019, we collected forest field measurements, including the position, species, height, and crown width of each tree in the field. A Blume-Leiss ALTImeter (Forestry Suppliers, Inc., Jackson, MS, United States) was used to measure tree heights trigonometrically (Sun et al., 2016). The crown lengths of each tree in the north–south (N–S) and east–west (E–W) directions were measured with a tape along the trunk in both perpendicular directions. Coupled with field measurements, we marked the position of each treetop that could be recognized by visual inspection in the aerial photographs. Although a manual measurement approach is inherently subjective, this method is considered to provide a reliable and effective source of information on the tree crown distribution and affords an auxiliary means for verifying our retrieved results. Partial aerial photographs taken from the sample plots provided by Figure 1A the tree nursery, Figure 1B the forest landscape area, and Figure 1C the mixed tree habitat are shown in Figure 1.

### Training Samples Comprised of Heightmaps Generated From Point Clouds

**FIGURE 2:**
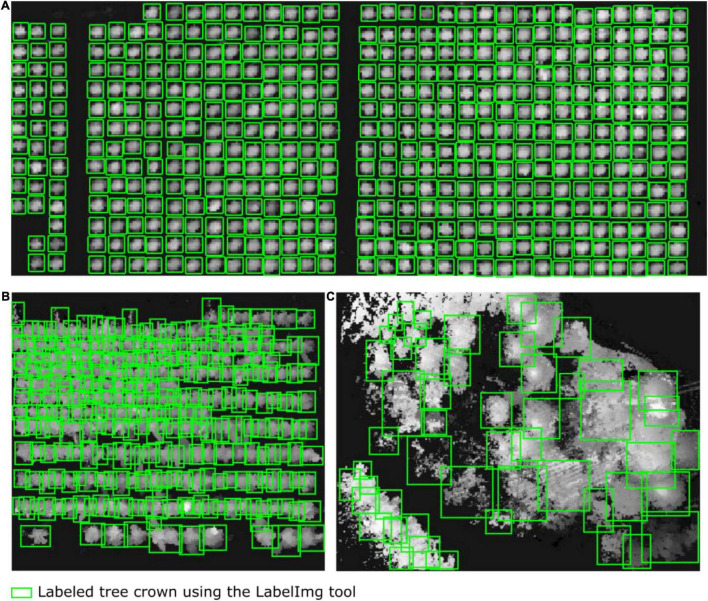
Diagrams showing some of the training samples manually labeled using the LabelImg tool. The tree crowns in the heightmaps were generated from the airborne LiDAR data of **(A)** the tree nursery, **(B)** the forest landscape area, and **(C)** the mixed tree habitat.

We first adopted a Gaussian filter (Xu et al., 2020) to remove noise and outliers from the point cloud data. Then, the point cloud data provided by the airborne LiDAR system were separated into ground points and non-ground points by using cloth simulation filtering (Qi et al., 2016). By orthographically projecting the non-ground point clouds, a planar raster (i.e., heightmap) was generated in the form of a DSM converted from point clouds; the raster comprised uniformly distributed and horizontal square grids (pixels) *c*_*i*_ of size *d* with the assigned elevation value equal to the highest elevation of all scanned tree points within each cell *c*_*i*_. Consequently, we rescaled the range of grid values in the DSM (heightmap) to Liu et al. (2017); i.e., we specified the value of each grid cell as the fraction relative to the maximum height of the current scanned points regarding the study forest plot. Because the average point density was approximately 130 points per square meter and the average point spacing across our studied forest plots was approximately 10 cm, we set the size d of the squared grid cell to 15 cm. This guaranteed at least three scanned points within one grid cell, thereby avoiding empty cells and preserving the detailed morphological features of the target forest canopy. Next, we used LabelImg to manually label 812, 703, and 754 trees (green boxes) in the heightmaps of the tree nursery, forest landscape area, and mixed tree habitat, respectively. Figure 2 shows some manually collected training samples at each of the three study sites.

### Augmenting the Training Data Using Three GAN Variants

Deep learning models always require a large amount of training data to optimize a massive number of parameters if the models are to learn how to extract high-quality features. GANs have made a dramatic leap in modeling the high-dimensional distributions of visual data and have shown remarkable success in synthesizing high-fidelity images and in generating stylized task-oriented training samples without additional manual annotation and device collection.

In this section, to generate visually appealing samples comprising tree crown heightmaps as supplementary training samples, we deliberately selected three advanced GANs, i.e., Cycle-GAN with unpaired image-to-image translation (Zhu et al., 2017), WGAN-GP with improved training (Gulrajani et al., 2017), and SinGAN (Shaham et al., 2019), and we addressed the conceptual differences between them.

#### Network Structure and Loss Function of Cycle-Consistent Generative Adversarial Networks

For CycleGAN, image-to-image translation is utilized to learn the mapping between the input images and output images using a training set of aligned image pairs. Many tasks, such as style transfer, object transfiguration, season transfer and photo enhancement, can be achieved. Here, we selected two sets of manually annotated images (each set containing 513 individual tree heightmaps) as the paired training data to generate another two sets of synthetic training samples to double the number of training samples. The loss function $Loss_{G}^{CycleGAN}$ of equation (1) is used to optimize the parameters of the two generators *G*_*A*→*B*_ and *G*_*B*→*A*_, which transfers one dataset (*x*_*A*_ or *x*_*B*_) to a new dataset [*G*_*A*→*B*_(*x*_*A*_) or *G*_*B*→*A*_(*x*_*B*_)] under the instructions of the semantic features of another training set (*x*_*B*_ or *x*_*A*_). This approach satisfies three criteria: (i) the generator takes its output data as the input data, and it can yield the same result; (ii) the output of the generator can confuse the corresponding discriminator; and (iii) the generator should follow backward cycle consistency, i.e., *G*_*B*→*A*_(*G*_*A*→*B*_(*x*_*A*_)) ≈ *x*_*A*_, where || ||_1_ denotes the 1-norm.


(1) $$\begin{array}{c} Loss_{G}^{CycleGAN}=5/n\cdot (||G_{A\to B}\left(x_{B}\right)-x_{B}|{|}_{1}\\ \;+||G_{B\to A}\left(x_{A}\right)-x_{A}|{|}_{1})\\ \;+{\left(D_{B}\left(G_{A\to B}\left(x_{A}\right)\right)-1\right)}^{2}\\ \;+{\left(D_{A}\left(G_{B\to A}\left(x_{B}\right)\right)-1\right)}^{2}\\ \;+10/n\cdot (||G_{B\to A}\left(G_{A\to B}\left(x_{A}\right)\right)-x_{A}|{|}_{1}\\ \;\;+||G_{A\to B}\left(G_{B\to A}\left(x_{B}\right)\right)-x_{B}|{|}_{1}) \end{array}$$

The loss functions $Loss_{D}^{CycleGAN}$ for two discriminators (i.e., *D*_*A*_ and *D*_*B*_), which explore the robust performance to discriminate between real (*x*_*A*_ or *x*_*B*_) and fake (*G*_*A*→*B*_(*x*_*A*_) or *G*_*B*→*A*_(*x*_*B*_)) samples, are determined as follows.


(2) $$\left\{ \begin{array}{c} Loss_{D_{A}}^{CycleGAN}={\left(D_{A}\left(x_{A}\right)-1\right)}^{2}+{\left(D_{A}\left(G_{B\to A}\left(x_{B}\right)\right)-0\right)}^{2}\\ Loss_{D_{B}}^{CycleGAN}={\left(D_{B}\left(x_{B}\right)-1\right)}^{2}+{\left(D_{B}\left(G_{A\to B}\left(x_{A}\right)\right)-0\right)}^{2} \end{array}\right.$$

**FIGURE 3:**
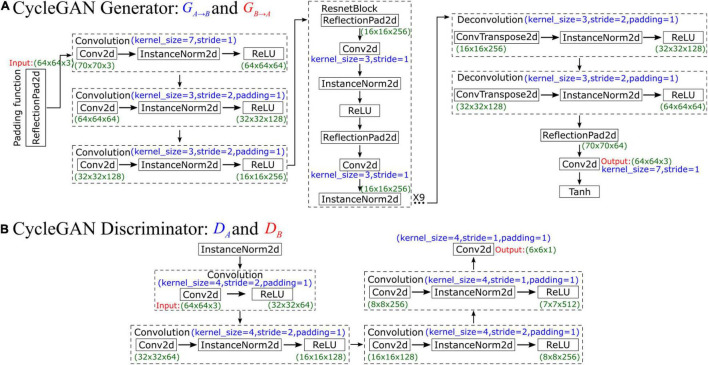
Schematic diagram showing the **(A)** generator and **(B)** discriminator of CycleGAN.

CycleGAN’s generator network comprises three parts, namely, an encoder, a converter, and a decoder, which are composed of three convolutional layers, nine residual blocks, and two fractionally strided convolutional layers. The network is illustrated in Figure 3A. First, the original input data size is 64×64×3. To increase the contributions of pixels along the borders of the original image, we use a padding function to expand the original data, and the input data size after padding is 70×70×3. After that, the encoder performs three convolutions, and the number of feature maps increases from 3 to 64, then to 128, and finally to 256. In each convolution, the InstanceNorm2d function is used for normalization during the evaluation, and ReLU is an activation function. After the convolutions are finished, the output data size is 16×16×256. As the training progresses deeper, the network uses ResnetBlock to avoid vanishing and exploding gradient problems. Therefore, the generator can achieve better performance because ResnetBlock adds skip connections based on simple forward propagation. However, ResnetBlock does not change the data size, so the output data size after 9 ResnetBlocks is still 16×16×256. Then, the decoder performs 2 deconvolutions, and the data are upsampled in size from 16×16×256 to 64×64×64. Finally, one last padding function and convolution function are used, and the final output data size is 64×64×3. The Tanh activation function is finally applied to make the final data comparable to the original data.

In CycleGAN’s discriminator, InstanceNorm2d is used for normalization during evaluation first, and the input data size is 64×64×3. Then, the network performs a convolution that obtains 64 feature maps and compresses the data size to 32×32×64. After that, InstanceNorm2d is added to perform three convolutions such that the number of feature maps increases from 64 to 512. The output data size is 7×7×512, which is also considered the input data size for the next convolution. Finally, after this last convolution, the final output data size is 6×6×1, which is a matrix. Each value in this matrix represents the true possibility of a receptive field in the image corresponding to a patch of the image. Unlike the discriminator networks of previous GANs, which use only one probability to judge the authenticity of the whole generated result, CycleGAN’s discriminator makes a judgment on each small patch. In other words, the discriminator performs a comparison between the real data and input data on 6×6 = 36 patches and normalizes their similarity to a value between 0 and 1. During the training process, CycleGAN calculates the arithmetic mean of this matrix to judge the difference from the real image. The network is illustrated in Figure 3B.

**FIGURE 4:**
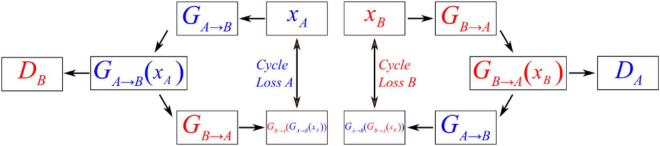
Schematic diagram showing the operating principles between the generator and discriminator of CycleGAN.

Figure 4 shows the operating principles between two generators and two discriminators in CycleGAN, i.e., *G*_*A*→*B*_, *G*_*B*→*A*_, *D*_*A*_, and *D*_*B*_, where the two generators have the same network structure as the discriminators. In the training process, generator *G*_*A*→*B*_ will perform convolutions on input data A to generate *G*_*A*→*B*_(*x*_*A*_). Then, this generated result will be carried into *D*_*B*_ to output a matrix. CycleGAN uses Markovian discriminator, that is, a discriminator makes convolutions to the input data, which are generated by the generator, and maps the input to a patch matrix. This process allows the discriminator to evaluate the results of the generator, and CycleGAN can learn the features of data B. After the network generates *G*_*A*→*B*_(*x*_*A*_), the result is also carried into *G*_*B*→*A*_ to generate *G*_*B*→*A*_(*G*_*A*→*B*_(*x*_*A*_)), which is used to calculate the cycle loss between *x*_*A*_ and *G*_*B*→*A*_(*G*_*A*→*B*_(*x*_*A*_)). This ensures that the final output bears a similarity to data A rather than only having features of data B. For data B, CycleGAN applies the same operations to achieve the generation of *G*_*B*→*A*_(*x*_*B*_), which is similar to data B but has the features of data A.

#### Loss Function and Network Structure of Wasserstein Generative Adversarial Networks + Gradient Penalty

Wasserstein generative adversarial networks + gradient penalty uses a 1-Lipschitz constraint coupled with a gradient penalty item to strengthen its discrimination performance. The improved loss functions $Loss_{G}^{WGAN-GP}$ and $Loss_{D}^{WGAN-GP}$ are shown in equations (3) and (4), respectively, where *x* is real data, *z* is random array data, *D* is the discriminator, *G*(z) denotes the generated fake samples, and *mean*() represents the computational average of all the elements in the input array. The third item on the right side of equation (3) denotes the gradient penalty item, which consecutively generates samples through linear interpolation between the real and generated data in each iterative step to drive the discriminator toward a better solution. The minimization of equation (4) allows the generator to deceive the discriminator.


(3) $$\begin{array}{c} Loss_{D}^{WGAN-GP}\\ \;=mean\left(D\left(G\left(\text{z}\right)\right)\right)-mean\left(D\left(x\right)\right)\\ \;+10\text{×}{\left({\left\|\frac{\partial \left(D\left(rand\cdot x+\left(1-rand\right)\cdot G\left(\text{z}\right)\right)\right)}{\partial \left(rand\cdot x+\left(1-rand\right)\cdot G\left(\text{z}\right)\right)}\right\|}_{2}-1\right)}^{2} \end{array}$$

(4) $$Loss_{G}^{WGAN-GP}=-mean\left(D\left(G\left(\text{z}\right)\right)\right)$$

**FIGURE 5:**
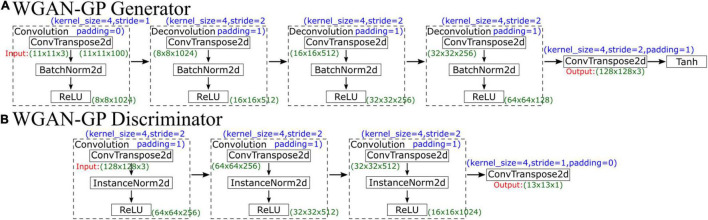
Schematic diagram showing the **(A)** generator and **(B)** discriminator of WGAN-GP.

WGAN-GP’s generator network contains five parts, which are illustrated in Figure 5A. The first part is a convolutional layer, followed by three deconvolutional layers and finally a simple convolution function. The first four parts each comprise a convolution function (ConvTranspose2d), a normalization function (BatchNorm2d, which is used for normalization during evaluation) and a ReLU activation function layer. All kernels are of size 4×4 with a stride of two except the stride of the first convolution, which is 1. The generator first increases the number of channels from 3 to 100, so the input data size is 11×11×100; after convolution, the size becomes 8×8×1024. Then, the network performs four deconvolutions, and the number of feature maps decreases from 1024 to 128, so the output data size is 64×64×128. Finally, through a simple convolution function, the final output data size is 128×128×3. Finally, a Tanh activation function is used to make the final data comparable to the original data.

The input in the discriminator is the output of the generator, so the input data size is 128×128×3. The discriminator network structure is similar to that of the generator, which has 4 parts, as illustrated in Figure 5B. The first three convolution layers comprise a convolution function, an instance normalization function, and an activation function. After convolution, the data size becomes 16×16×1024. Then, there is a deconvolution function with a stride of 1, and the final output data size is 13×13×1; the final output is not a single probability but a matrix. Similar to CycleGAN, each value in the matrix represents a true possibility of a receptive field in the image. During the training process, WGAN-GP uses the “mean” function to calculate the average value of this matrix to judge the difference from the real image.

#### Loss Function and Network Structure of SinGAN

SinGAN can learn from a single natural image and contains a pyramid of fully convolutional GANs to capture the internal feature distribution of various scale patches within the image. Moreover, SinGAN can generate high-quality and diverse samples that carry the same visual content as the input image. Three items constitute the loss function of the SinGAN discriminator, which is shown in equation (5). In each iteration step, the fake images yielded by generator *G* are based on the joint input as z_*s*_ + *x*_*s* + 1_, where *s* is the sample scale with a smaller value representing a coarser scale through the upsampling operation and vice versa for larger values, *x*_*s* + 1_ represents an upsampled version of the image from the finer scale *s* + 1, *z*_*s*_ denotes the random noise at scale *s*, and *D* is the discriminator. Similar to $Loss_{D}^{WGAN-GP}$ in WGAN, a gradient penalty exists in the discriminator loss function $Loss_{D}^{SinGAN}$ that ensures a specific set of input noise maps at the *s*th scale coupled with the generated image at the coarser scale *s* + 1 to satisfy the conditions of generating the original images at the *s*th scale as much as possible. Usually, the input image is transformed into eight scales from coarse to fine, and the generator and discriminator work at each scale to propagate the results to the next (finer) scale with injected random noise to optimize the neural connection weights. Formula (6) shows the loss function of the SinGAN generator, whose aim is to generate real images to confuse the discriminator. Additional noise ${\text{z}}_{s}^{\prime }$ is incorporated with a random noise z_*s*_ to achieve different style transfers and foreground object texture transfers to match different backgrounds.


(5) $$\begin{array}{c} Loss_{D}^{SinGAN}=mean\left(D\left(G\left({\text{z}}_{s}+x_{s+1}\right)\right)\right)-mean\left(D\left(x_{s}\right)\right)\\ \;+0.1\text{×}{\left({\left\|\frac{\begin{array}{c} \partial (D(rand\cdot x_{s}+\left(1-rand\right)\\ \cdot G\left({\text{z}}_{s}+x_{s+1}\right))) \end{array}}{\begin{array}{c} \partial (rand\cdot x_{s}+\left(1-rand\right)\\ \cdot G\left({\text{z}}_{s}+x_{s+1}\right)) \end{array}}\right\|}_{2}-1\right)}^{2} \end{array}$$

(6) $$Loss_{G}^{SinGAN}=mean{\left(G\left({\text{z}}_{s}+{\text{z}}_{s}^{\prime }\right)-x_{s}\right)}^{2}$$

**FIGURE 6:**
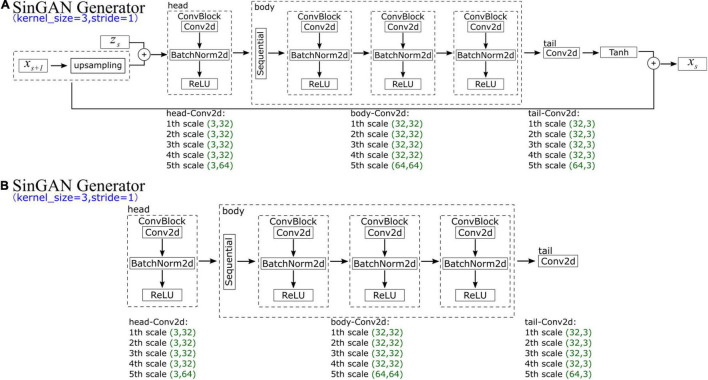
Schematic diagram showing the **(A)** generator and **(B)** discriminator of SinGAN.

In SinGAN’s generator, at each scale *s*, the input data comprise *x*_*s* + 1_ (an upsampled image that is generated by the previous generator *G*_*s* + 1_) and corresponding random noise *z*_*s*_. Each generator scale contains five convolution layers, which can be divided into three parts (the head has one convolution, the body has two convolutions, and the tail has one convolution). In the head and body convolutions, the structure is the same, comprising a convolution function (ConvTranspose2d), a normalization function (BatchNorm2d, which is used for normalization during evaluation) and a ReLU activation function layer. In contrast, the tail part contains only a convolution function. However, the parameters of the input and output channels of the convolution change every five scales; we list the parameters of the first five scales in Figure 6A. The generated data *x*_*s*_ are the convoluted result added to the input upsampled image *x*_*s* + 1_.

SinGAN’s discriminator, whose structure is depicted in Figure 6B, has an adversarial goal. The network has the same structure as SinGAN’s generator, which also comprises five convolution layers and is divided into three parts (the head has one convolution, the body has two convolutions, and the tail has one convolution). Moreover, the parameters of the input and output channels of the convolution change every five scales along with the generator.

**FIGURE 7:**
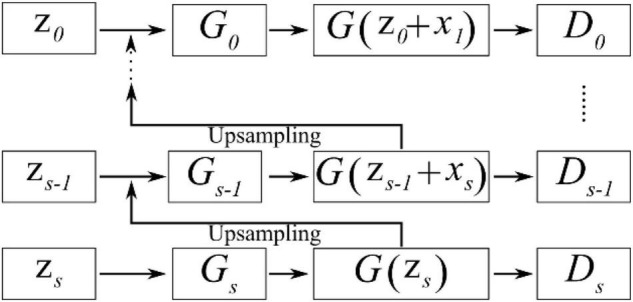
Schematic diagram showing the pyramid structure in SinGAN’s generator and discriminator.

SinGAN’s network structure is similar to the pyramid structure shown in Figure 7 and is based on the idea of upsampling from coarse to fine. That is, the size of the effective patch decreases from the bottom to the top of the pyramid, and upsampling occurs at each scale. The input data at the coarsest scale are only random noise z_*s*_; except at this scale, the generator generated *G*(z_*s*−1_ + *x*_*s*_) through noise z_*s*−1_ and upsampled data *x*_*s*_, and the output data will be carried into the discriminator for a comparison with the real data. Similar to that of CycleGAN, SinGAN’s discriminator is also a Markovian discriminator; thus, the output data are a matrix, and each value in this matrix represents the true possibility of a 11×11 receptive field in the image. During the training process, SinGAN calculates the arithmetic mean of this matrix to judge the difference from the real image. From the coarsest scale to the finest scale (from *G*_*s*_ to *G*_0_), the discriminator’s receptive field sizes are all 11×11. Because different scales have different input data sizes but the receptive field size is the same, amazing effects are generated. At the coarsest scale, the patch size is 1/2 of the size of the image; thus the GAN network can learn the global structure of the image. As the scale becomes finer, SinGAN can gradually add details that were not generated at the previous scales.

### Improved YOLO-v4 Network

**FIGURE 8:**
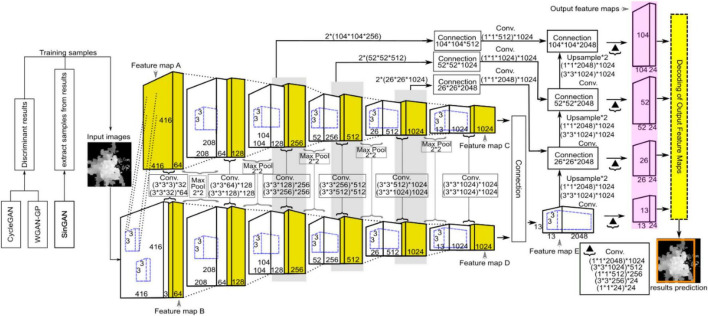
Schematic diagram showing the network structure of YOLO for ITC segmentation, where CycleGAN, WGAN-GP, and SinGAN were used for training data argumentation. YOLO was adopted for ITC detection from the heightmaps of the studied forest canopy.

As an end-to-end detection system, the entire network structure of YOLO is shown in Figure 8. Different from the original YOLO-v4 network structure, we use the PANet structure on four valid feature layers, which increases the scale compared with the original three scales. This means that we have an extra output feature map. YOLO-v4 utilizes global reasoning for the whole image to predict the relevant information of all the objects, mainly including the prediction of the bounding boxes and corresponding confidence. The YOLO-v4 detection processes are as follows: First, the appropriate bounding box priors are automatically generated by clustering the labeled bounding boxes using K-means clustering, and the number of clusters is set as *B*, which means that the number of anchor boxes is *B*. This value guarantees that the model is simple while achieving high recall. Then, the image is input into the YOLO-v4 network for feature extraction, and the feature map with a size of *M*×*M* is output. The network predicts bounding boxes for each grid cell of the output feature map and predicts the confidence and location coordinates $\left(\hat{x},\hat{y},\hat{w},\hat{h}\right)$ of each bounding box. Then, it constrains the four coordinates to obtain the center coordinates (*p*_*x*_, *p*_*y*_) and the value of width and height (*p*_*w*_, *p*_*h*_) of the predicted box relative to the image. *l*_*x*_ and *l*_*y*_ are the confidence scores, which represent the offset of the current cell grid relative to the upper left corner of the image, and ${\tilde{w}}_{\mu }$ and ${\tilde{h}}_{\mu }$ are the width and height of the anchor boxes, respectively. In formulas (7) and (8), the sigmoid function σ is used to limit *x* and *y* in the current grid, which facilitates convergence, and the formulas for calculating the bounding box coordinates are as follows.


(7) $$p_{x}=\sigma \left(\hat{x}\right)+l_{x}$$

(8) $$p_{y}=\sigma \left(\hat{y}\right)+l_{y}$$

(9) $$p_{w}={\tilde{w}}_{\mu }e^{\hat{w}}$$

(10) $$p_{h}={\tilde{h}}_{\mu }e^{\hat{h}}$$

Second, YOLO-v4 obtains the confidence of the predicted boxes by determining whether the center of an object is in each grid cell. If it does not exist, the confidence value is zero; otherwise, the confidence value is the intersection over union (*IoU*) of the bounding box prior and the ground truth, where *IoU* is the ratio of their intersection area to their union area. The range of *IoU* is between 0 and 1, where 0 means that two boxes do not overlap at all and 1 indicates that the two boxes are equal., i.e., $IoU_{tru}^{pri}$. For $IoU_{tru}^{pri}$, we set the threshold to 0.5. If $IoU_{tru}^{pri}\leq 0.5$, the prediction score should be ignored; otherwise, only when the $IoU_{tru}^{pri}$ value of a bounding box prior and the ground truth are greater than that of any other bounding box prior is the object score of the corresponding predicted box 1.

Finally, YOLO-v4 chooses an independent logical classifier for class prediction. When the method is applied to our dataset, YOLO predicts a 3D tensor for each scale of output, *M*×*M*×[3×(4 + 1 + 1)], which represents the four parameter values of prediction, that is, three scales, four coordinates, one object, and one class.

In our experiment, the purpose was to identify ITCs from a whole heightmap, and we expected to find more appropriate anchor boxes through a clustering algorithm in this small target detection problem, which was helpful for improving the average precision and speed of small target detection. In the original YOLO-v4 model, K-means clustering, which is an unsupervised algorithm, is used to obtain the anchor boxes to predict the coordinates of the bounding boxes. *K*-means aims to partition *n* observations into *B* clusters, in which each observation belongs to the cluster with the nearest mean, serving as a prototype of the cluster.

At the beginning, *K*-means obtains the sizes of all the bounding boxes and then randomly selects *B* cluster centroids, and these cluster centroids have a width ${\tilde{w}}_{\mu }$ and height ${\tilde{h}}_{\mu }\left(\mu =1,\ldots ,B\right)$. Then, the following process is repeated until convergence: For the number of *n* bounding boxes in the training dataset, we seek the manual annotation using ImageLabel and obtain a series of bounding box widths as $w_{i}^{obj}$ and heights as $h_{i}^{obj}\left(i=1,\ldots ,n\right)$. Then, the cluster it should belong to is calculated, and for each cluster μ(μ = 1,…, *B*), the centroid of the cluster is recalculated. The objective function of *K*-means is as follows.


(11) $$E=\sum_{i=1}^{n}\sum_{\mu =1}^{B}{\left\|\left(w_{i}^{obj},h_{i}^{obj}\right)-\left({\tilde{w}}_{\mu },{\tilde{h}}_{\mu }\right)\right\|}^{2}$$

In formula (11), *n* is the number of sample bounding boxes in the training dataset, *B* is the number of clusters, $\left(w_{i}^{obj},h_{i}^{obj}\right)$ are the coordinates of the bounding boxes, and $\left({\tilde{w}}_{\mu },{\tilde{h}}_{\mu }\right)$ is the cluster centroid. This formula describes the tightness of samples in the cluster around the mean of the cluster. The similarity of samples in the cluster increases as the value of *E* decreases. In summary, *K*-means is a cyclic process of finding a more suitable cluster centroid and assigning samples to the closest cluster centroid until the objective function converges.

As mentioned above, in the original YOLO-v4 clustering algorithm (*K*-means), distance is the only factor that affects the clustering results; thus, other attributes are not considered. If the cluster contains noise samples or isolated samples that are far from the data sample space, a large fluctuation arises in the calculation of the cluster center. This fluctuation greatly impacts the mean value calculation and even makes the cluster center seriously deviate from the dense area of the cluster sample, resulting in substantially biased results. In addition, *K*-means needs to specify the number of clusters in advance before processing the data, and the designation of this number is highly subjective. Here, to select more suitable anchor boxes for small target detection, we sought to optimize the clustering algorithm and adopted Mean Shift, which is a non-parametric, feature-space mathematical analysis technique for locating the maximum of a density function. A detailed description of Mean Shift is as follows.

Mean Shift uses kernel density estimation, which is the most popular density estimation method. In our experiment, the anchor boxes have two properties: length and width. For samples in the training dataset, we seek the manual annotation using ImageLabel and obtain a series of bounding boxes with their approximate sizes, i.e., $w_{i}^{obj}$ and $h_{i}^{obj}\left(i=1,\ldots ,\tilde{n}\right)$. In addition, we initialize a center box ${\tilde{X}}_{\mu }$ whose width is ${\tilde{w}}_{\mu }$ and whose height is ${\tilde{h}}_{\mu }\left(\mu =1,2,3\ldots \right)$. Hence, we implement kernel density estimation in two-dimensional space, and the expression is as follows.


(12) $$\hat{f}\left({\tilde{X}}_{\mu }\right)=\frac{1}{\tilde{n}H^{2}}\sum_{i=1}^{\tilde{n}}CK\left(\frac{{\tilde{X}}_{\mu }-X_{i}}{H}\right)$$

In formula (12), *H* is the bandwidth, which is the parameter to be specified, $\tilde{n}$ is the number of data points in set $\tilde{S}$, *K*(*X*) is the kernel function, $X_{i}=\left(w_{i}^{obj},h_{i}^{obj}\right)$, and ${\tilde{X}}_{\mu }=\left({\tilde{w}}_{\mu },{\tilde{h}}_{\mu }\right)$. $\tilde{S}$ is a set comprising $\tilde{n}$ bounding boxes *X*_*i*_ that satisfy formula (13), which indicates that the distances between all the bounding boxes *X*_*i*_ in $\tilde{S}$ and the center box ${\tilde{X}}_{\mu }$ are less than a given threshold *ξ*. The normalization constant *C*, which makes the kernel function *K*(*X*) integrate to one, is assumed to be strictly positive.


(13) $$\begin{array}{c} \tilde{S}=\{\left(w_{i}^{obj},h_{i}^{obj}\right)|\left[\left(w_{i}^{obj},h_{i}^{obj}\right)-\left({\tilde{w}}_{\mu },{\tilde{h}}_{\mu }\right)\right]\\ \;{\left[\left(w_{i}^{obj},h_{i}^{obj}\right)-\left({\tilde{w}}_{\mu },{\tilde{h}}_{\mu }\right)\right]}^{T}\leq \xi^{2}\} \end{array}$$

Different kernel functions *K*(*X*) correspond to different transformations of the original sample data. The common profile of the kernel function can be classified into four types: linear kernel, polynomial kernel, radial basis function kernel and sigmoid kernel. The specific mathematical expressions for these types are as follows:


(14) $$\begin{array}{c} \text{Linear}\mathrm{kernel}:\;K_{L}\left(x\right)=x^{T}x+c\\ \text{Polynomial}\mathrm{kernel}:K_{P}\left(x\right)={\left(\gamma x^{T}x+c\right)}^{d}\\ \mathrm{RBF}\mathrm{kernel}:\;K_{R}\left(x\right)=\exp\left(-\frac{x^{T}x}{2\sigma^{2}}\right)\\ \mathrm{Sigmoid}\mathrm{kernel}:K_{S}\left(x\right)=\tanh\left(\gamma x^{T}x+c\right) \end{array}$$

For our study, bounding boxes only have two features, i.e., width and height. This number of features is relatively small compared to the larger number of features in the training samples. The linear kernel *K*_*L*_(*x*) employs dot products to optimize the efficiency of resolving the problem, and the sound predictive performance of *K*_*L*_(*x*) is achieved when the feature number of the samples is larger. However, this is not suitable for our bounding box classification that only includes two features. The computational complexity of the polynomial kernel (Xu et al., 2022) is relatively high, and it may suffer from numerical instability because a detrimental tendency beyond control is prone to occur when γ*x^T^x* + *c* < 1, *K*_*P*_(*x*) trends to zero with increasing *d*, which is in contrast to the opposite case when γ*x^T^x* + *c* > 1, *K*_*P*_(*x*) tends to infinity. Hence, reasonable parameter assignment for the three parameters of *a*, *c*, and *d* is comparatively not easy. Sigmoid kernel *K*_*S*_(*x*) is typically suitable for neural networks but is computationally expensive. RBF kernel *K*_*R*_(*x*) is a popular kernel function (Fan et al., 2022) used in various kernelized learning algorithms, which maps a single vector to a vector of higher dimensionality with the superior classification performance for the larger number of training samples with fewer features, similar to the input data of the bounding box properties.

According to (12) and (14), the kernel density estimation (12) can be rewritten as follows.


(15) $$\hat{f}\left({\tilde{X}}_{\mu }\right)=\frac{C}{\tilde{n}H^{2}}\sum_{i=1}^{\tilde{n}}K\left({\left\|\frac{{\tilde{X}}_{\mu }-X_{i}}{H}\right\|}^{2}\right)$$

The process of Mean Shift is to calculate the vector $\tilde{M}$ and then update the position of the center point to make the center of the circle move in the direction of the maximum density in the dataset. The derivative of formula (15) is required to calculate vector $\tilde{M}$, and the derivative function is shown below.


(16) $$\hat{\nabla }f\left({\tilde{X}}_{\mu }\right)=\frac{2C}{\tilde{n}H^{4}}\sum_{i=1}^{\tilde{n}}\left({\tilde{X}}_{\mu }-X_{i}\right)K^{\prime }\left({\left\|\frac{{\tilde{X}}_{\mu }-X_{i}}{H}\right\|}^{2}\right)$$

Then, if we simplify the equation even further, we can obtain formula (17).


(17) $$\begin{array}{c} \nabla \hat{f}\left({\tilde{X}}_{\mu }\right)=\frac{2C}{\tilde{n}H^{4}}\left[\sum_{i=1}^{\tilde{n}}-K^{\prime }\left({\left\|\frac{{\tilde{X}}_{\mu }-X_{i}}{H}\right\|}^{2}\right)\right]\\ \;\times \left[\frac{\sum_{i=1}^{\tilde{n}}X_{i}K^{\prime }\left({\left\|\frac{{\tilde{X}}_{\mu }-X_{i}}{H}\right\|}^{2}\right)}{\sum_{i=1}^{\tilde{n}}K^{\prime }\left({\left\|\frac{{\tilde{X}}_{\mu }-X_{i}}{H}\right\|}^{2}\right)}-{\tilde{X}}_{\mu }\right] \end{array}$$

Only if the second half of formula (17) equals 0 can $\hat{\nabla }f=0$. Therefore, vector $\tilde{M}$ can be described as follows.


(18) $$\tilde{M}=\frac{\sum_{i=1}^{\tilde{n}}X_{i}K^{\prime }\left({\left\|\frac{{\tilde{X}}_{\mu }-X_{i}}{H}\right\|}^{2}\right)}{\sum_{i=1}^{\tilde{n}}K^{\prime }\left({\left\|\frac{{\tilde{X}}_{\mu }-X_{i}}{H}\right\|}^{2}\right)}-{\tilde{X}}_{\mu }$$

After obtaining and applying $\tilde{M}$ to the current center point ${\tilde{X}}_{\mu }$, we obtain the new center point ${\tilde{X}}_{\mu }^{new}=\left({\tilde{w}}_{\mu }^{new},{\tilde{h}}_{\mu }^{new}\right)$ and repeat the above process. With each iteration, the current center point moves toward the new center point. Finally, ${\tilde{X}}_{\mu }^{new}$ becomes the new cluster center, which can be described as formula (19).


(19) $${\tilde{X}}_{\mu }^{new}={\tilde{X}}_{\mu }+\tilde{M}=\frac{\sum_{i=1}^{\tilde{n}}X_{i}K^{\prime }\left({\left\|\frac{{\tilde{X}}_{\mu }-X_{i}}{H}\right\|}^{2}\right)}{\sum_{i=1}^{\tilde{n}}K^{\prime }\left({\left\|\frac{{\tilde{X}}_{\mu }-X_{i}}{H}\right\|}^{2}\right)}$$

After several iterations, when the distance between the center point and the point where the gradient of kernel density estimate (12) is zero and less than the threshold *ξ*, the iteration ends, and we obtain the final cluster center ${\tilde{X}}_{\mu }^{final}=\left({\tilde{w}}_{\mu }^{final},{\tilde{h}}_{\mu }^{final}\right)$ to represent the highest-probability density center.

When the first round of iteration ends and the final cluster center ${\tilde{X}}_{1}^{final}=\left({\tilde{w}}_{1}^{final},{\tilde{h}}_{1}^{final}\right)$ is calculated, another center box ${\tilde{X}}_{2}$ is set up from the beginning. If ${\tilde{X}}_{2}$ is close to ${\tilde{X}}_{1}^{final}$, ${\tilde{X}}_{2}$ drifts to ${\tilde{X}}_{2}^{final}$, which coincides with ${\tilde{X}}_{1}^{final}$ after the Mean Shift algorithm. In this case, ${\tilde{X}}_{2}^{final}$ cannot be defined as a new highest-probability density center. Only if the distance between ${\tilde{X}}_{2}$ and ${\tilde{X}}_{1}^{final}$ is relatively far, which means ${\tilde{X}}_{2}$ is in another density region, will ${\tilde{X}}_{2}$ drift to a truly new highest-probability density center ${\tilde{X}}_{2}^{final}$; this means that Mean Shift calculates a new highest-probability density center.

Because of the disadvantages of *K*-means, noisy samples or isolated samples in the cluster may seriously affect the clustering results, and the number of categories is highly subjective. However, Mean Shift can analyze the information of bounding boxes through $w_{i}^{obj}$ and $h_{i}^{obj}$, which are manually annotated, and find the center boxes with the highest-probability density. As a result, Mean Shift can filter out noise samples or isolated samples and identify the number of categories automatically, which can improve the clustering results to provide more appropriate anchor boxes for future detection.

### Training Data Augmentation and Testing for Improved YOLO-v4 Network

We utilized the labeled images as training samples to train CycleGAN, WGAN-GP, and SinGAN. The CycleGAN, WGAN-GP, and SinGAN models trained on the augmented data were used to produce additional outputs of the samples, generating 1187, 1326, and 1263 supplementary training samples for the tree nursery, forest landscape area, and mixed tree habitat, respectively. In conjunction with the manually labeled images, all the training samples were brought into the improved YOLO-v4 network to find the appropriate weights of the neural connections. In addition, we extracted nine sample plots from the tree nursery, forest landscape, and mixed tree habitat and manually labeled 59, 84, 333, 65, 45, 82, 96, 76, and 117 trees planted in these nine sample plots as the sample trees for testing.

Before training, we conducted transfer learning based on the pretrained model by using the convolutional weights of the pretrained model trained on the Common Objects in Context (COCO) dataset (Belongie, 2014) to set the initial weights. Moreover, the dimensions (width × height) of the input images (i.e., heightmaps) for the training set were resized to the defaults of416×416. For the training process, we trained the YOLO network for approximately 70,000 iterations. We used a batch size of 64 and a momentum of 0.9 for gradient-based optimizers with a decay of 0.0005. The initial learning rate was set to 0.001 for fast convergence. As the training process proceeded, the final learning rate decreased to 0.0001 for numerical stability. The total training time was approximately 24 h.

The testing process of the YOLO network included three main steps: (1) taking the selected nine sample plots from the three forest plot types as the testing sets and the corresponding heightmaps generated from the scanned points of these sample plots; (2) resizing these heightmaps as 416×416 and bringing them into the YOLO network for feature extraction and target recognition; and (3) analyzing the output feature maps and verifying the predicted bounding boxes of the tree crowns by reference field data.

During testing, three different detection metrics were employed: the number of true positives (*TP*), which is the actual number of trees that are correctly detected; the number of false positives (*FP*), which is the number of incorrectly detected (non-existent) trees (that is, the commission error); and the number of false negatives (*FN*), which is the number of undetected actual trees (that is, the omission error). Here, *TP* + *FP* represents the total number of trees detected by our method, whereas the total number of actual trees is expressed as *TP* + *FN*.

The detection efficiency of the model is the main factor affecting the test results. To evaluate the performance of our method, this paper selects the precision (*p*), recall (*r*), and (*F*_1_) score (*F*_1_) as the evaluation indexes. Here, *p* represents the number of trees correctly detected divided by the total number of trees detected by the model; *r* represents the number of trees correctly detected by the model divided by the actual number of trees, that is, the detection rate; and *F*_1_ represents the harmonic mean between *p* and *r* (Gao et al., 2020). The closer the values of *p*, *r*, and *F*_1_ are to 1, the greater the efficiency and the better the performance of the YOLO network. Briefly, *p*, *r*, and *F*_1_ are defined by the following equations.


(20) $$p=\frac{TP}{TP+FP}$$

(21) $$r=\frac{TP}{TP+FN}$$

(22) $$F_{1}=2\times \frac{p\times r}{p+r}$$

### Overlapping Tree Segmentation Using the Fitted Elliptical Paraboloids

**FIGURE 9:**
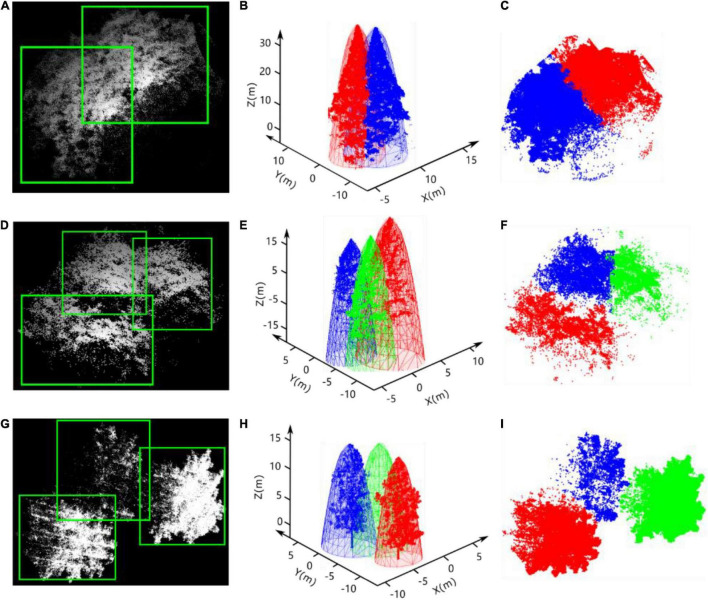
Results of tree detection by improved YOLO-v4, the elliptic paraboloid fitting of tree crowns, and the segmentation of overlapping trees. As shown in **(A,D,G)**, the white points and green rectangular boxes represent the point cloud of adjacent trees and the bounding boxes, respectively. The paraboloid fitting results of each adjacent tree crown and the point cloud of each tree are shown in **(B,E,H)**. **(C,F,I)** Are the results of the segmentation of points in the intersecting area based on our method.

After the bounding boxes for all of the tree crowns in the heightmaps are predicted by YOLO, intersecting areas always exist between adjacent bounding boxes, even those placed correctly around many neighboring trees, as shown in Figures 9A,D,G. Hence, the affiliation of the points in the intersecting area to the specific tree crown must be determined. According to the biophysical characteristics of trees, tree crowns usually have approximately regular geometrical shapes and smooth peripheries caused by the transport of nutrients from the roots to distal tips and gravitropism (Duchemin et al., 2018). An elliptic paraboloid, an open surface generated by rotating a parabola about its axis, was adopted here to fit each adjacent tree crown based on the points in the non-intersecting regions of each bounding box. Then, the distances between the points in the intersecting area and the fitted elliptical paraboloids of each adjacent tree crown were taken as a criterion to determine the affiliation of points in the intersecting area, as shown in Figures 9C,F,I.

Here, we adapted the least squares method (Savitzky and Golay, 1964) to calculate the parameters of the optimal paraboloid surface of the τth tree *S*_τ_ based on the points $p_{i}^{tree_{\tau }}\left(x_{i}^{tree_{\tau }},y_{i}^{tree_{\tau }},z_{i}^{tree_{\tau }}\right)$ in the non-intersecting area of the bounding box predicted by the improved YOLO-v4 network.

According to the geometric features of tree crowns, we set the fitted paraboloid to be open downward, and its vertex was located at the corresponding treetop ${\hat{p}}^{tree_{\tau }}\left({\hat{x}}^{tree_{\tau }},{\hat{y}}^{tree_{\tau }},{\hat{z}}^{tree_{\tau }}\right)$ with *a* and *b* equal to the half-crown width in the N–S and E–W directions, respectively. The specific formula is defined as follows.


(23) $$f\left(x,y,z\right)=-\frac{{\left(x-{\hat{x}}^{tree_{\tau }}\right)}^{2}}{a^{2}}-\frac{{\left(y-{\hat{y}}^{tree_{\tau }}\right)}^{2}}{b^{2}}+{\hat{z}}^{tree_{\tau }}-z\left(x,y\right)=0$$

Then, the least squares method was employed here to seek the best-fitting paraboloid for the points $p_{i}^{tree_{\tau }}\left(x_{i}^{tree_{\tau }},y_{i}^{tree_{\tau }},z_{i}^{tree_{\tau }}\right)$ by minimizing the sum of the distances between the points and the fitted paraboloid surface, i.e., making the following equation obtain the smallest value.


(24) $$\begin{array}{c} \underset{a,b}{\arg\min}f\left(a,b\right)=\sum_{i=1}^{\psi }[-\frac{{\left(x_{i}^{tree_{\tau }}-{\hat{x}}^{tree_{\tau }}\right)}^{2}}{a^{2}}\\ \;-\frac{{\left(y_{i}^{tree_{\tau }}-{\hat{y}}^{tree_{\tau }}\right)}^{2}}{b^{2}}+{\hat{z}}^{tree_{\tau }}-z_{i}^{tree_{\tau }}]{}^{2} \end{array}$$

In formula (24), ψ represents the total number of points of the τth tree in the non-intersecting area of the bounding box predicted by the YOLO network. To calculate the optimal parameters *a* and *b*, which is an unconstrained extremum problem of a binary function with *a* and *b* as independent variables, the derivatives of formula (24) with respect to *a* and *b* are calculated. The mathematical expressions are as follows.


(25) $$\left\{ \begin{array}{c} \frac{\partial f}{\partial a}=2\sum_{i=1}^{n}\left[-\frac{{\left(x_{i}^{tree_{\tau }}-{\hat{x}}^{tree_{\tau }}\right)}^{2}}{a^{2}}-\frac{{\left(y_{i}^{tree_{\tau }}-{\hat{y}}^{tree_{\tau }}\right)}^{2}}{b^{2}}+{\hat{z}}^{tree_{\tau }}-z_{i}^{tree_{\tau }}\right]\\ \left[2\frac{{\left(x_{i}^{tree_{\tau }}-{\hat{x}}^{tree_{\tau }}\right)}^{2}}{a^{3}}\right]=0\\ \frac{\partial f}{\partial b}=2\sum_{i=1}^{n}\left[-\frac{{\left(x_{i}^{tree_{\tau }}-{\hat{x}}^{tree_{\tau }}\right)}^{2}}{a^{2}}-\frac{{\left(y_{i}^{tree_{\tau }}-{\hat{y}}^{tree_{\tau }}\right)}^{2}}{b^{2}}+{\hat{z}}^{tree_{\tau }}-z_{i}^{tree_{\tau }}\right]\\ \left[2\frac{{\left(y_{i}^{tree_{\tau }}-{\hat{y}}^{tree_{\tau }}\right)}^{2}}{b^{3}}\right]=0 \end{array}\right.$$

Notably, the solution to equation set (25) is not unique. When multiple solutions exist, multiple solutions of function *f* exist. Here, we choose the values of *a* and *b* corresponding to the smallest values as the optimal parameters. After calculating the optimal parameters *a* and *b*, the fitted paraboloid surface determined by formula (24) for each tree crown can be drawn. The schematic diagrams are shown in Figures 9B,E,H.

After obtaining the fitted elliptic paraboloids for the adjacent tree crowns, the next task is to calculate the shortest distance $dist_{S_{\tau }}^{P^{{}_{inter}}}$ between the points $p_{j}^{inter}$ in the intersecting area and *S*_τ_. For this purpose, we sought the points $p_{l}^{e}\left(x_{l}^{e},y_{l}^{e},z_{l}^{e}\right)$ on the elliptic paraboloids closest to $p_{j}^{inter}$ with the shortest distance, i.e., the normal vector of the paraboloid at point $p_{l}^{e}$ should be parallel to the vector between $p_{l}^{e}$ and $p_{j}^{inter}$. Then, we used equation set (26) to calculate the coordinates of $p_{l}^{e}$ for each point $p_{j}^{inter}$ in the intersecting area.


(26) $$\left\{ \begin{array}{c} f(x_{l}^{e},y_{l}^{e},z_{l}^{e}=0\\ \frac{\partial f}{\partial y}\cdot \left(x_{l}^{e}-x_{j}^{inter}\right)-\frac{\partial f}{\partial x}\cdot \left(y_{l}^{e}-y_{j}^{inter}\right)=0\\ \frac{\partial f}{\partial z}\cdot \left(y_{l}^{e}-y_{j}^{inter}\right)-\frac{\partial f}{\partial y}\cdot \left(z_{l}^{e}-z_{j}^{inter}\right)=0 \end{array}\right.$$

In the above equation, ⋅ represents the dot product, and the solution, namely, the corresponding $p_{l}^{e}\left(x_{l}^{e},y_{l}^{e},z_{l}^{e}\right)$ on the fitted elliptic paraboloid with the shortest distance to $dist_{S_{\tau }}^{P^{{}_{inter}}}=|p_{l}^{e},p_{j}^{inter}|$, can be obtained. In a group of several adjacent trees, a point within the intersecting areas of the boundary boxes defined by YOLO can be determined by comparing the shortest distance from the point to each fitting paraboloid, i.e., the smallest magnitude of the distance from the point to the fitted paraboloid of the τth tree corresponding to the affiliation of the point to the τth tree. The segmentation results of the point cloud in the intersecting areas are shown in Figures 9C,F,I.

## Results

### Evaluation of the You Only Look Once Detection Effect

**FIGURE 10:**
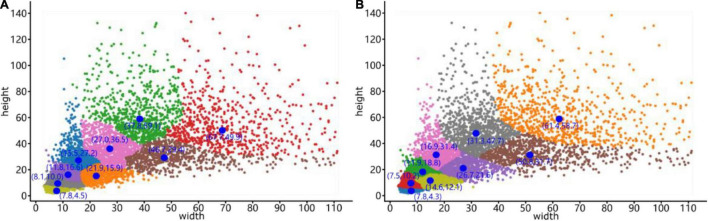
Clustering results generated by the **(A)** *K*-means clustering algorithm and **(B)** Mean Shift clustering algorithm. The blue dots represent the cluster centers, and the coordinates are next to these cluster centers, while the *x*-coordinate is the width and the *y*-coordinate is the height.

To verify the feasibility of the optimized clustering approach, we used K-means and Mean Shift to cluster bounding boxes on the same dataset. The dataset contained 9 sample plots belonging to the three forest plot types (812, 703, and 754 trees were manually annotated in the heightmaps of the tree nursery, forest landscape area, and mixed tree habitat, respectively) for a total of 2269 tree samples. Figure 10 shows the clustering results generated by these two clustering algorithms.

The differences and anchor box detection results after clustering optimization are compared in Table 1. In the table, the average precision is calculated by the *IoU* of the bounding box prior and the ground truth, which is the ratio of their intersection area to their union area, and the calculation method is described in section “Improved YOLO-v4 Network.” The cluster centers for the sizes of anchor boxes obtained by the Mean Shift algorithm significantly improve the target detection performance, with the detection speed being 2.46 frames per second (FPS) higher than that of the original YOLO-v4 network. In addition, the average detection precision is increased by 1.75%, reaching 91.42%. After demonstrating the effectiveness of our optimization method, a mean shift clustering algorithm was used for ITC detection testing. The testing process and evaluation metrics are presented in Section “Individual TreeCrown segmentation using a deep learning model.”

**TABLE 1:** Comparison of the average detection precision and FPS between YOLO trained on K-means and YOLO trained on Mean Shift on the same manually annotated ITC dataset (all trained on the overall training samples of the three study sites).

Clustering algorithm	Cluster centers for the sizes of anchor boxes	Average precision (%)	Speed detection (FPS)
K-means	(7.8,4.5),(8.1,10.0), (11.8,16.6), (15.5,27.2),(21.9,15.9), (27.0,36.5), (37.8,59.1),(46.7,29.4), (67.7,49.9)	89.67	41.35
Mean Shift	(7.5,10.2),(7.8,4.3), (11.9,18.8), (14.6,12.1),(16.9,31.4), (26.7,21.6), (31.3,47.7),(50.8,31.7), (61.4,58.7)	91.42	43.81

### Results of the Training Process

**FIGURE 11:**
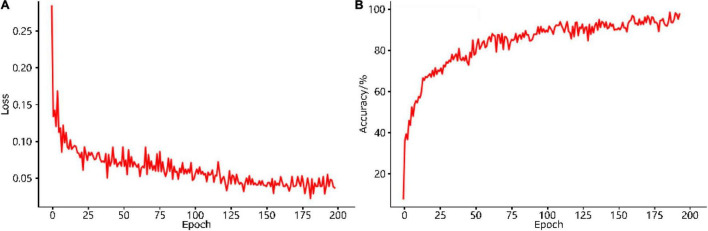
**(A)** Loss curve and **(B)** accuracy curve of the improved YOLO-v4 model in the training process.

The training loss curve of the improved YOLO-v4 model is shown in Figure 11A. The loss decreases rapidly in the first 50 epochs and gradually stabilizes after 150 epochs, with a final loss of approximately 0.04. The time and rate of convergence of the loss curve depend mainly on the selection of an appropriate learning rate (Zhang et al., 2020c). At the beginning of training, a higher initial learning rate needs to be set due to the lack of known information. As training progresses, the learning rate must be reduced such that the loss function can converge to the optimal value more smoothly. Our training obtained a small final loss, which shows that the error between the predicted value and the ground-truth value of the network is small and that the model exhibits good performance.

The training accuracy curve of the improved YOLO-v4 model is shown in Figure 11B. The accuracy increased rapidly and exceeded 80% in the first 50 periods, then it steadily increased until reaching nearly 98% after 200 periods; this indicates that our classifier makes very small prediction errors.

### Synthetic Tree Crown Heightmap Generation by CycleGAN, WGAN-GP, and SinGAN

**FIGURE 12:**
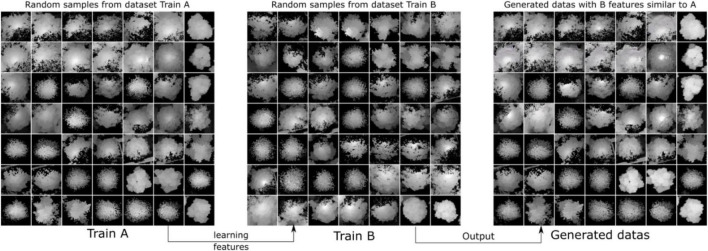
Diagrams showing some of the training samples and generated samples. We collected two sets of training samples in CycleGAN: Train A and Train B. The purpose was to generate style transfer images that are similar to Train A but have the features of Train B.

CycleGAN was used to generate synthetic heightmaps of ITCs. We randomly collected two sets of training samples (Train A and Train B, each set containing 513 random individual tree heightmaps from three forest plot types). As each of these heightmaps is unique, stylistic differences exist between these two sets. CycleGAN captures special characteristics from Train B and determines how these characteristics can be translated into Train A, which is in the absence of any paired training examples. As a result, we can generate heightmaps using special learned features from Train B, and the style transfer-generated heightmap results can be used in the YOLO model. The training and generated synthetic tree crown heightmaps are shown in Figure 12.

**FIGURE 13:**
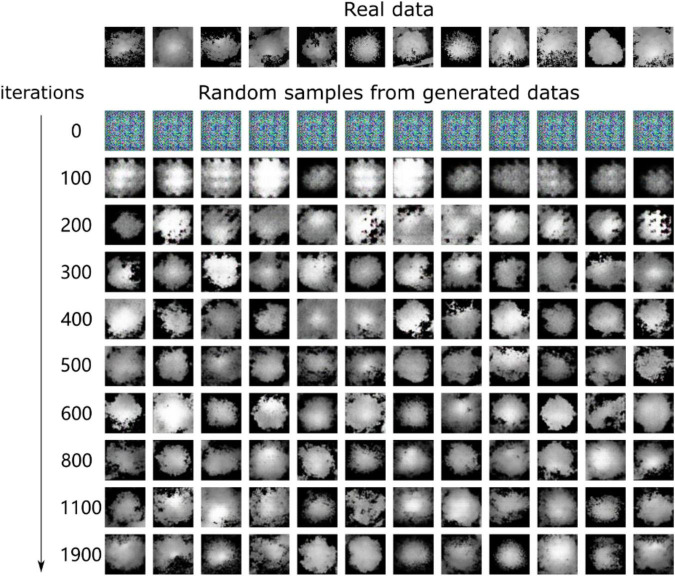
Heightmaps generated using WGAN-GP with an increasing number of iterations. At the 0th, 100th, and 200th iterations, the generated data distributions are very different from the real data distributions. However, as the training process continues, the generator can produce heightmaps of tree crowns with the same or nearly the same quality and successfully fool the discriminator in WGAN-GP.

To augment the training sets for the YOLO model, we considered generating more “different” heightmaps. WGAN-GP was used to generate more synthetic heightmaps of ITCs and the training dataset containing 1264 random individual tree heightmaps from three forest plot types. The trained parameters of the WGAN-GP models during the training stage were saved every 100 iterations as the number of training iterations increased. Additionally, synthetic images of tree crowns were generated based on the training parameters every 100 iterations and compared with the expected target images. After the generative process of WGAN-GP, we chose 10 sets of training parameter files and the corresponding 10 sets of generated heightmaps (including the 0th iteration). When the generator uses the training parameters at the 100th and 200th iterations (which do not satisfy the loss convergence for the neural network), the generated image textures are completely random and contain much noise. As the training progress continued and the number of iterations reached 300 and 400, the generator learned certain basic features of the real data, and some generated heightmaps already resembled the real data. Then, the quality of the synthetic images was improved by considering additional training iterations. We chose the 1100th and 1900th iterations to show that the evolution results of the generated images looked very realistic and were very close to the expected image. The generated synthetic tree crown heightmaps with an increasing number of training iterations are shown in Figure 13.

**FIGURE 14:**
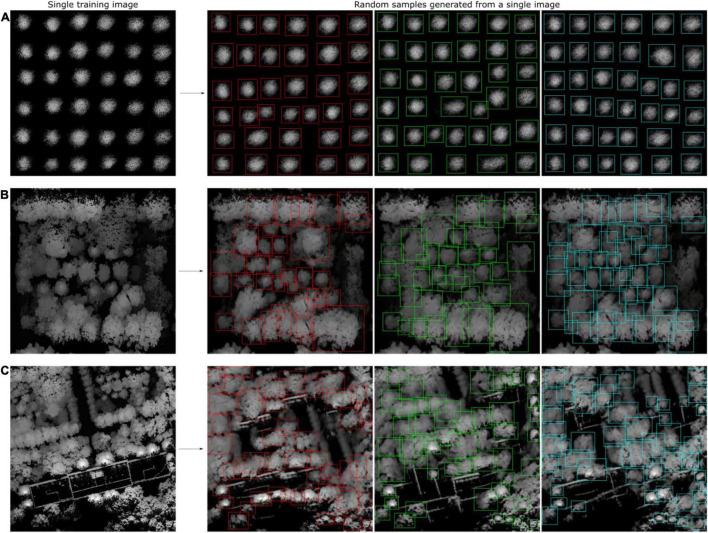
SinGAN was used to generate the heightmaps of a forest plot. Diagrams showing the generated synthetic heightmaps of **(A)** the tree nursery, **(B)** the forest landscape area, and **(C)** the mixed tree habitat. Although a portion of the generated image is slightly fuzzy, most of the tree crowns can be visually identified. Thus, these images with the tree crowns in the synthetic heightmaps manually labeled using the LabelImg tool can be used as new training sets.

After using CycleGAN and WGAN-GP to generate synthetic heightmaps of ITCs, SinGAN, an unconditional generative model that can be learned from a single natural image, was used to generate synthetic heightmaps of a large area. SinGAN can generate high-resolution images from a forest plot. In total, 47 relatively large heightmap samples containing clear tree crowns were selected from three forest plot types to serve as training datasets for SinGAN. The generator learned an increasing number of characteristics of the training images as training scale increased. After 10 scales, the generated heightmaps had the same aspect ratio as the original image, and three generated samples are shown in Figure 14, revealing that in all these cases, the generated samples depict new realistic structures and configurations of objects while preserving the visual content of the training image. Due to SinGAN’s multiscale pipeline, the structures at all scales, from the global arrangement of big tree crowns to the fine textures of the seedlings, are nicely generated.

### Individual TreeCrown Segmentation Using a Deep Learning Model

**FIGURE 15:**
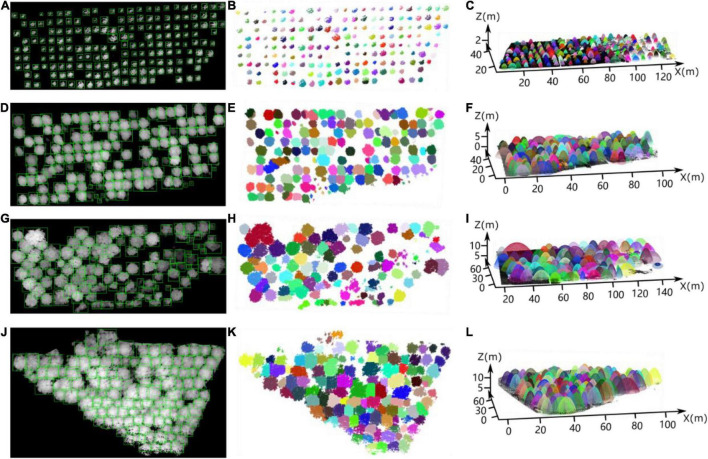
Individual tree crown detection results *via* our deep learning network for the partial testing set of heightmaps for the **(A)** nursery, **(D,G)** forest landscape area, and **(J)** mixed tree plantation. **(B,E,H,K)** Correspond to **(A,D,G,J)**, respectively, which intuitively display the ITC detection results for the four study sites. **(C,F,I,L)** Show the results of elliptic parabolic fitting for each tree in the four plots, corresponding to **(A,D,G,J)**, respectively.

We selected three forest plots from each of the three forest plots for the test set, yielding a total of nine forest plots. In the test set, 476, 192, and 289 sample trees were from the nursery, forest landscape area and mixed tree plantation, respectively. After testing the test set using the small target detection framework of improved YOLO-v4, 432, 166, and 238 trees were detected correctly, respectively, 50, 36, and 55 non-existent tree crowns were detected by mistake, and 66, 38, and 60 trees were missed. The tree crown detection results (green boxes) of improved YOLO-v4 in the test sets of the Figure 15A tree nursery, Figures 15D,G forest landscape, and Figure 15J mixed forest habitat are shown in Figure 15. Figures 15B,E,H,K visually represent the ITC detection test results, and each detected tree is identified by different colors. In addition, we performed elliptic parabolic fitting for each tree, and the results are shown in Figures 15C,F,I,L.

Table 2 lists the ITC detection results for the 9 sample plots belonging to the three forest plot types. The *p*-values of the nine sample plots ranged from 0.75 to 0.87, and the average value of *p* for the nursery (0.86) was higher than that for the forest landscape area (0.80) and mixed forest habitat (0.78). Considerable differences in the *r* values (ranging from 0.75 to 0.85) relative to the omission error were also observed among the nine sample plots. Moreover, compared to the range of *F*_1_ values calculated for the forest landscape areas (0.80–0.82) and mixed tree plantations (0.75–0.83), the *F*_1_ values of the sample plots of the nursery were all ≥ 0.84.

**TABLE 2:** Accuracy assessment of ITC detection by our deep learning method for the three forest plot types in the nursery, forest landscape area and mixed tree plantation.

Study site	Number of sample trees			TP	FP	FN	*p*	*r*	F1
	Training		Test (Sample plot)						
Nursery	M/G	812/1187	59 (plot 1)	51	8	8	0.86	0.83	0.84
			84 (plot 2)	77	10	14	0.85	0.85	0.85
			333 (plot 3)	304	32	44	0.87	0.84	0.85
Forest landscape area	M/G	703/1326	65 (plot 4)	58	14	12	0.81	0.83	0.82
			45 (plot 5)	37	8	8	0.82	0.82	0.82
			82 (plot 6)	71	14	18	0.78	0.80	0.80
Mixed tree plantation	M/G	754/1263	96 (plot 7)	82	18	14	0.82	0.85	0.83
			76 (plot 8)	65	13	16	0.75	0.80	0.75
			117 (plot 9)	91	24	30	0.77	0.75	0.76
Overall	M/G	2269/3776	957	836	141	164	0.81	0.84	0.82

*TP, number of correctly detected actual trees; FP, number of detected non-existent trees (that is, the commission error); FN, number of undetected actual trees (that is, the omission error); p, number of correctly detected trees divided by the total number of trees detected by the model; r, the number of trees correctly detected by the model divided by the actual number of trees; F_1_, harmonic mean of p and r; M, number of manually labeled ITCs from heightmaps; G, number of generated synthetic ITCs from heightmaps by CycleGAN, WGAN-GP, and SinGAN.*

Although the nursery contained twice as many trees as either the mixed tree plantation or the forest landscape area, the numbers of commission errors and omission errors in the nursery were less than those in the mixed tree plantation and forest landscape area. A reasonable explanation for this situation is that the canopy environments of the forest landscape area and mixed tree plantation are complex due to the high degree of tree species diversity, large variations in tree ages, and different growth statuses of the trees, whereas the trees in the nursery have simple horizontal and vertical structures. Therefore, the performance of the deep learning network in the nursery is better than that in the mixed tree plantation and forest landscape areas. To test this interpretation, we analyzed the comparison between the linear regression models for the predicted canopy size and field measurement data at the three study sites.

First, we transformed the cardinal directions of the heightmaps of the studied forest plots with north at the top and east at the right.

Then, after predicting the width (vertical) and length (horizontal) of the bounding boxes by YOLO on each heightmap and determining the affiliation of the points in the intersecting regions, the crown lengths in the N–S and E–W directions were obtained.

**FIGURE 16:**
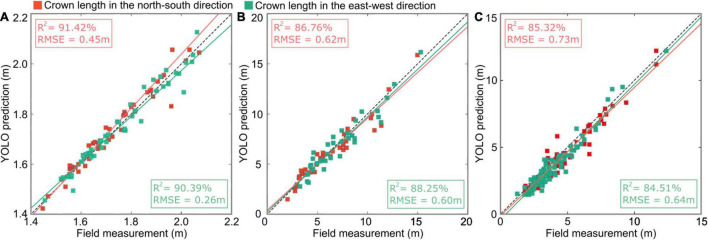
Scatter plots showing the relationship of the predicted crown lengths from improved YOLO-v4 versus the field measurement data in **(A)** sample plot 1 of the tree nursery, **(B)** plot 5 of the forest landscape, and **(C)** plot 7 of the mixed tree plantation, where the red squares represent the crown lengths in the N–S direction and the green squares represent the crown lengths in the E–W direction. The red and green lines are the fitted lines for the N–S direction and E–W direction using least squares regression, respectively.

Figure 16 shows the linear regression results based on the canopy lengths in the N–S and E–W directions predicted by our deep learning method and the field measurement data at the three study sites. The linear regression models of the predicted crown widths and field data in the three study sites were analyzed with two statistical indicators: the coefficient of determination *R*^2^ and the root-mean-square error (RMSE). The largest *R*^2^ (90.91 ± 0.51%) and smallest RMSE (0.36 ± 0.10 m) were achieved in the nursery (Figure 16A plot 1) due to the uniform planting arrangement of small, homogeneous trees. Relatively lower *R*^2^ values (87.51 ± 0.75%) and larger RMSEs (0.61 ± 0.01 m) were obtained in the forest landscape area (Figure 16B plot 5) due to the existence of well-designed plants with varying heights, which formed beautiful scenery with a multilayered forest structure. However, certain parts of the shorter tree crowns in the subcanopy layer may be obstructed by neighboring taller trees from a bird’s-eye view. The smallest *R*^2^ (84.82 ± 0.41%) and relatively large RMSEs (0.68± 0.05 m) were obtained in the mixed tree plantation (Figure 16C plot 7) due to the anisotropic crown shape and interlacing branches of adjacent trees.

The linear regression results of canopy lengths in the N–S and E–W directions predicted by the deep learning network for the above three plots indicate that the complexity of the canopy environment affects the prediction accuracy of the deep learning network.

## Discussion

By effectively extracting and analyzing the feature information from a large number of training samples, deep learning provides technical assistance for the actualization of intelligent systems in the fields of self-driving cars (Li et al., 2020), target recognition (Jin et al., 2021) and tracking, and automatic voice recognition (Ma et al., 2021). In recent years, methods that combine remote sensing data with deep learning techniques have been increasingly applied to solve problems in forestry, such as individual tree segmentation (Wang et al., 2019), tree species classification (Hamraz et al., 2019), and crown information interpretation (Wu et al., 2020). In this study, the deep learning-based improved YOLO-v4 network combined with a heightmap converted from airborne LiDAR data was first used to detect ITCs in different types of forest plots.

### Feasibility of Our Method

Aerial photography provides high-resolution remote sensing images (Song et al., 2021) and is often used to map, manage, and analyze tree distributions (Xu et al., 2019). However, the captured tree crowns consistently exhibit considerable differences in appearance due to varying capture positions between UAV-loaded cameras and the target trees. In addition, solar illumination directions, atmospheric turbidity, weather conditions and the varying phenological periods of tree crowns reduce the certainties of tree crown recognition. Airborne LiDAR facilitates acquisition the vertical structure of the upper forest canopy at multiple scales with variable spot sizes (Phua et al., 2017). Although the development of LiDAR technology has enabled studies *via* the acquisition of small- to medium-scale regional data, the efficacy is still affected by various factors, e.g., mutually occluded vegetative elements, intermediate and suppressed trees hidden below the upper forest canopy, and the diverse geometrical features of tree crown appearances diminishing the uniform presentation of tree crowns. To overcome the restrictions of aerial photography, we considered using the YOLO deep learning model based on a heightmap directly generated from airborne LiDAR data. When employed in combination with some refinement of this deep learning method and trained by GAN-generated augmented datasets, a high ITC segmentation accuracy can be achieved without external objective factors.

Individual tree crown in the nursery, forest landscape area, and mixed tree plantation environments were detected using our deep learning method with 86.8, 81.4, and 79.9% overall recall, respectively (the data is the overall recall of three plots from each forest plot type, which is not in Table 2), indicating that our method can attain a relatively stable ITC detection rate in different forest environments. The ITC detection rate tends to decrease with increases in the tree species diversity, planting density and canopy structural complexity. Compared with other automated methods (Hu et al., 2014) used to delineate ITCs (72–74% detection rate) in high-density LiDAR data, our deep learning method displayed a pronounced enhancement in its tree crown detection ability. Moreover, compared with a previous study using different airborne remotely sensed data [i.e., Multidetector Electro-Optical Imaging Scanner (MEIS)-II data and IKONOS satellite image data] to identify individual trees (Corresponding et al., 2004), our method has similar or greater accuracy. Since our method exhibited good robustness and scalability in different types of forest plots and achieved relatively high accuracy in the automatic and real-time detection of tree crowns, the proposed method based on a deep learning framework has potentially wide applications in forestry and related fields (Ma et al., 2022).

In this study, the degree of complexity of the forest canopy structure increased from the nursery to the forest landscape area and then to the mixed tree plantation. In an open system, gaps always exist between tree saplings, and the lateral and vertical growth of small trees at the initial growth stage with immature tree crown are rarely obscured by the adjacent tree crowns at roughly equal heights. In addition, the small degree of species diversity, the lack of understory trees in the sample plots and the minimal differences in tree crown shapes also yielded a favorable impact on the testing of trees in the nursery. Therefore, compared with the forest landscape area and mixed tree plantation, our method achieved the highest overall values of the three indexes, namely, *p*(0.82), *r*(0.87), and *F*_1_ (0.84), for a single study site when assessing the nursery testing samples.

For the various tree species living in well-pruned and maintained landscapes and mixed tree plantations, strong lateral branches with multifoliate clumps usually appear, which causes spurious peaks, with the surrounding area having a declining height and a tendency to be mistakenly detected as an isolated tree crown. Complete crown surfaces of morphological vagueness are difficult to extract with respect to trees with overlapping and interlacing branches as well as blurred crown drip lines such that the number of tree crowns may be overestimated from multiple clumped tree crowns during the detection process of deep learning. In the forest landscape area and mixed tree plantation, omission errors were caused mainly by the understory vegetation and suppressed trees located between adjacent trees forming interlocked tree crowns. During the point cloud data acquisition for the dense forest, only part of the laser pulse can reach the lower layer of the canopy through the forest gaps due to the occlusion caused by the vegetation elements in the emergent and canopy layers, which deteriorates the forest information description from the middle and lower canopy point cloud data (Almeida et al., 2019). Hence, we selected only trees taller than 3 m for analyzing and evaluating the ITC detection efficacy in the forest landscape area and mixed tree plantation.

In addition, the pixel values of a grayscale heightmap range from 0 to 1, corresponding to the *z* values of point clouds in each grid (pixel). Usually, 0 is the lowest height representing the ground, and 1 is the highest height value coinciding with the treetop of the tallest tree in the plot. If the suppressed trees below the general level of the forest canopy have relatively small heights and exhibit an inconspicuous dark gray color contrasting with the dark color of ground points, they possibly represent indistinct visual texture features and impair the deep learning recognition ability. An image processing strategy for color contrast enhancement, i.e., histogram equalization, is recommended for heightmaps to strengthen the hidden image features of dwarf tree crowns.

### Comparison of Detection Results Using Different Methods

A comparative experiment was conducted to explore the performance of our method versus the traditional watershed method (Hu et al., 2014).

**FIGURE 17:**
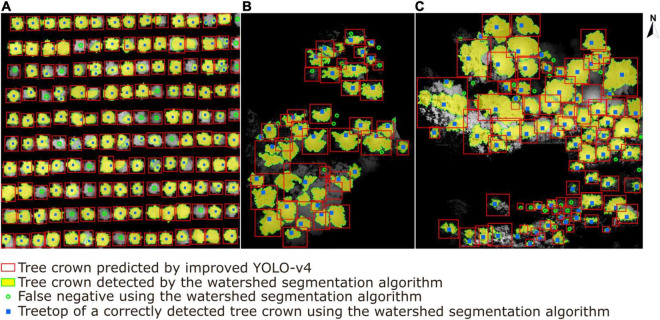
A comparison results of individual tree crown segmentation shown in **(A)** the tree nursery, **(B)** the forest landscape area, and **(C)** the mixed tree habitat using the watershed segmentation algorithm (yellow areas with extracted green boundaries) versus our deep learning algorithm (red boxes). The blue squares represent the correctly detected treetops of tree crowns by watershed segmentation, and the green hollow dots represent the tree crowns missed by watershed segmentation.

The results of the watershed segmentation algorithm and our deep learning approach are presented in Figure 17. In total, 212 trees were detected correctly (solid blue square) in sample plots, whereas 32 non-existent trees were mistakenly detected as trees. In addition, 54 trees, especially sub-canopy trees, were not detected (green hollow dots) when using the watershed segmentation algorithm. Although the watershed segmentation algorithm shows good and relatively stable effects compared to other traditional canopy detection algorithms under different environments, some parameters, i.e., the size and variance of the smoothing template or the threshold for water expansion control, depend upon calibration for specific conditions (Yun et al., 2021). The deep learning method shows better performance in generality and robustness with respect to high tree species diversity and different forest plot types (Chen et al., 2021). The results show that the detection rate of ITCs by the watershed segmentation algorithm is 79.7%, which is 3.9% lower than that of our deep learning network. An extrapolation of these findings is that as tree species diversity and planting density in the sample plots increase, an increase in this gap of tree crown detection accuracy between two methods will appear.

In the second experiment, to explore the differences between training the YOLO network on the dataset of manually labeled ITCs and training the network on a dataset enhanced with the GAN-generated synthetic ITCs, we compared the detection performances after testing the improved YOLO-v4 detection model on three forest plot types. As all the network models we experimented with used the same initial weights and hardware conditions, the dataset was the only difference.

Table 3 shows a comparison between the detection results of the YOLO network trained on the manually labeled dataset and those of the YOLO network trained on a dataset enhanced with the GAN-generated synthetic dataset, where the manually labeled dataset consisted of 2269 training sample trees and 340 test sample trees from the three types of forest plots, and the enhanced dataset consisted of 3776 training sample trees and the same 340 test sample trees as the manually labeled dataset. We assessed the detection accuracy and speed of the two YOLO networks with *p*, *r* and FPS, as FPS is a common measure for detecting the speed of object detection methods based on deep learning.

**TABLE 3:** Comparison results between the detection accuracy of YOLO trained on a manually labeled dataset and that of YOLO trained on an augmented GAN-generated synthetic dataset on the same testing samples (all trained on the overall training samples of the three study sites).

Training dataset	Number of sample trees		*p*	*r*	FPS
	Train	Test			
Manually labeled	2269	340	0.73	0.79	23
generated Synthetically + manually labeled	6405	340	0.81	0.84	38

In our experiments, training on the dataset enhanced with the GAN-generated synthetic dataset achieved superior performance, surpassing that achieved by training on manually labeled datasets in terms of both accuracy and speed. In terms of accuracy, the *p* and *r* values of the enhanced dataset outperformed those manually labeled by approximately 0.08 and 0.05, respectively. On our test set, the speed of YOLO trained on the enhanced dataset outperformed that of YOLO trained on the manually labeled dataset by 15 FPS.

## Conclusion

Our results show the effectiveness of the proposed deep learning object detection algorithm based on airborne LiDAR data at identifying ITCs from the heightmaps generated from point cloud data. Coupled with the synthetic training samples generated by CycleGAN, WGAN-GP, and SinGAN to augment the training sets, the deep learning network of the YOLO-v4 model was adopted to detect ITCs and calculate the corresponding crown widths of individual trees from heightmaps. In addition, we optimized the clustering algorithm in the YOLO-v4 network by adopting Mean Shift to replace K-means and proposed a method based on elliptic paraboloid fitting to determine the affiliation of the points in the intersecting regions between adjacent bounding boxes generated by the improved YOLO-v4 network for crown width estimation. The algorithm was validated by the test sets from three different types of forest plots (i.e., a tree nursery, a forest landscape area, and a mixed tree plantation), achieving the successful detection of 86.8, 81.4, and 79.9% of the tree crowns, respectively, in the three different test sets. The tree crown detection accuracy obtained in this study was slightly higher than that reported by previous studies. Therefore, our algorithm can quickly and accurately detect ITCs from various types of forest plots containing multiple tree species. Our method has pioneering potential for the small target detection capacity of deep learning networks to detect ITCs from heightmaps and affords heuristic perspectives guiding the development of deep learning techniques for forest point cloud analysis.

## Data Availability Statement

The raw data supporting the conclusions of this article will be made available by the authors, without undue reservation.

## Author Contributions

CS and TY contributed to the conception and design of the study. CH and HZ performed the statistical analysis. CS and CH wrote the first draft of the manuscript. BC, FA, and LW wrote sections of the manuscript. TY and CH contributed to the acquisition of data and critical revision of the manuscript for important intellectual content. All authors contributed to manuscript revision and read and approved the submitted version.

## Conflict of Interest

The authors declare that the research was conducted in the absence of any commercial or financial relationships that could be construed as a potential conflict of interest.

## Publisher’s Note

All claims expressed in this article are solely those of the authors and do not necessarily represent those of their affiliated organizations, or those of the publisher, the editors and the reviewers. Any product that may be evaluated in this article, or claim that may be made by its manufacturer, is not guaranteed or endorsed by the publisher.
